# Antidepressant Use and Risk of Suicidal Behavior in Older Persons With Depression: A Cohort Study in Hong Kong

**DOI:** 10.1093/geroni/igab046.1706

**Published:** 2021-12-17

**Authors:** Yi Chai, Hao Luo, Kenneth K C Man, Wallis C Y Lau, Ian C K Wong

**Affiliations:** 1 The University of Hong Kong, Hong Kong, Hong Kong; 3 University College London, London, England, United Kingdom

## Abstract

Background: Depression is highly prevalent in older adults and requires treatment. However, debate persists on whether antidepressant use is associated with an elevated risk of suicidal behavior. This study aims to examine the short- and long-term risk of suicidal behavior by various classes of antidepressants in older persons with depression. Methods: Persons aged 40 years and above and received a clinical diagnosis of depression between January 1, 2001, and December 31, 2016 were identified from the Clinical Data Analysis and Reporting System in Hong Kong. The risk of suicidal behavior in persons who were prescribed antidepressants was compared with persons who were not prescribed any antidepressant drugs. Antidepressants were classified as tricyclic and related antidepressant drugs (TCAs), selective serotonin reuptake inhibitors (SSRIs), noradrenergic and specific serotonergic antidepressants (NaSSAs), serotonin–norepinephrine reuptake inhibitors (SNRIs) and others. Incidence and adjusted hazard ratio (aHR) of subsequent self-harm and suicide within one-year and the whole study period were estimated by age groups. Results: A total of 34,927 persons aged 40-64 years, and 19,300 persons aged 65+ years were included. In the younger age group, the highest short-term and long-term risks were found in others (aHR, 2.33; 1.02-5.34) and NaSSAs (2.88; 2.15-3.86), respectively. In the older age group, no significant association was observed between antidepressant use and suicidal behavior across all antidepressant classes. Conclusion: The self-harm and suicide associated risks vary across antidepressant classes and age groups. Cautions are always needed for antidepressant prescriptions.

